# Evaluation of the Level of Agreement Between Clinical Diagnosis and Two Cephalometric Analyses: Cephalometric Analysis for Orthognathic Surgery (COGS) and Soft Tissue Cephalometric Analysis (STCA)

**DOI:** 10.1155/ijod/8655040

**Published:** 2025-03-14

**Authors:** Ankita Lohia, Siddarth Shetty, Amoli Singh, Shravan Shetty, Ashith M. V.

**Affiliations:** Department of Orthodontics and Dentofacial Orthopaedics, Manipal College of Dental Sciences Mangalore, Affiliated to Manipal Academy of Higher Education, Manipal, Karnataka, India

**Keywords:** cephalometrics, COGS, diagnostic accuracy, skeletal malocclusion, STCA, surgical intervention

## Abstract

**Introduction:** Hard tissue analysis, such as cephalometric analysis for orthognathic surgery (COGS), defines the nature of existing skeletal discrepancies but is incomplete in providing information concerning the facial form and proportions of the patient. The soft tissue cephalometric analysis (STCA) accounts for the soft tissue drape, which, however, is subject to significant individual, gender, and age variation.

**Aims and Objectives:** The purpose of the study was to evaluate the conformance of the diagnostic inferences derived from two cephalometric analyses, COGS and STCA, to the clinical diagnosis of experienced clinicians.

**Material and Methods:** Lateral cephalograms of 120 patients were traced for parameters previously diagnosed by an oral surgeon and an orthodontist. Corresponding variables were taken from two analyses, COGS and STCA, defining the (1) position of the maxilla, (2) position of the mandible, (3) growth pattern, (4) upper and lower lip prominence, (5) severity of skeletal malocclusion, and (6) need for surgical intervention. The inferences derived cephalometrically were compared to the clinical diagnosis.

**Results:** Kappa analysis was used to compare the agreement of inferences derived from COGS and STCA with clinical diagnosis. A *p*-value less than 0.016 was considered significant. The agreement of both analyses with clinical diagnosis was significant and fair when the position of the mandible and intermaxillary jaw relationship was considered. COGS showed better agreement for both. COGS additionally showed fair agreement with clinical diagnosis for growth patterns too. STCA showed fair agreement with clinical diagnosis when the need for surgical intervention was evaluated. For all other parameters, the agreement was poor for both analyses.

**Conclusion:** COGS proved to have better diagnostic accuracy than STCA, except in predicting the need for a surgical treatment plan, where STCA appeared better.

**Clinical Significance:** The findings provide significant insights that may improve the accuracy of diagnosis and decision-making in orthodontic and surgical interventions, ultimately aiding clinicians in selecting the most appropriate treatment protocols.

## 1. Introduction

Successful management of malocclusion begins with accurate diagnosis and comprehensive treatment planning [1]. The introduction of cephalometrics in orthodontics has revolutionized diagnostic protocols, enabling the development of over a hundred analyses to understand skeletal and dental relationships better. Since its inception, cephalometry has become indispensable for orthodontic diagnosis and treatment planning. However, relying solely on cephalometric parameters without considering individual variability, particularly in soft tissues, may compromise treatment outcomes [2].

Historically, different cephalometric approaches have focused on specific dentoalveolar structures. Dr. Angle emphasized achieving normal occlusion through tooth movement to address facial esthetics, while Tweed [3] prioritized mandibular incisor position. Downs [4] analyzed profile imbalances using hard tissue measurements, and Holdaway [5] highlighted the influence of soft tissue contour in defining facial esthetics. Hard tissue analyses, such as the cephalometric analysis for orthognathic surgery (COGS), specifically cater to maxillofacial surgery cases by using hard tissue landmarks and measurements that can be modified through surgical procedures. However, the limitation of such analyses lies in their inability to fully account for the influence of soft tissue on facial esthetics [2].

Soft tissue cephalometric analysis (STCA) by Arnett et al. [6] introduced a paradigm shift by emphasizing soft tissue measurements for orthodontic and orthognathic planning. This analysis accounts for variables such as soft tissue thickness at key anatomical sites, which significantly influence facial convexity and profile. Recent studies have underscored the variability of soft tissue thickness and its impact on the perception of skeletal discrepancies, especially in Class II and Class III malocclusion cases requiring surgical intervention. Borzabadi-Farahani et al. [7] highlighted key cephalometric characteristics of patients with severe Class II and Class III malocclusions, emphasizing the importance of integrating both hard and soft tissue assessments. These findings emphasized increased ANB angles, mandibular retrognathia in Class II cases, and mandibular prognathia with maxillary retrusion in Class III cases, highlighting the need for tailored orthognathic treatment approaches [7].

These considerations reiterate that treatment planning based solely on cephalometric norms is inadequate due to inherent interindividual variability. Clinical examination remains the cornerstone of diagnosis, with profiles ideally assessed in a natural head position, centric relation, and relaxed lip posture. Mankad et al. [8] found only 40% agreement on treatment planning when they analyzed 10 patients using five popular cephalometric analyses.

The current study aims to evaluate the conformity of inferences derived from COGS and STCA with clinical diagnoses by experienced clinicians, particularly in cases of severe skeletal malocclusion.

## 2. Materials and Methods

### 2.1. Study Design and Settings

This is a cross-sectional study conducted using the lateral cephalograms of 120 patients.

Good-quality lateral cephalograms were taken in the natural head position with soft tissue metallic markers and a plumb line on the film to indicate true vertical. Patients between the ages of 18 and 35 were included in the study. High-quality extraoral photographs of the patients were mandatory. Patients with any anomaly, airway obstruction, TMJ disorders, muscular disorders, prior orthodontic treatment, or systemic, or congenital disease were excluded from the study.

### 2.2. Lateral Cephalograms

**Figure 1:**
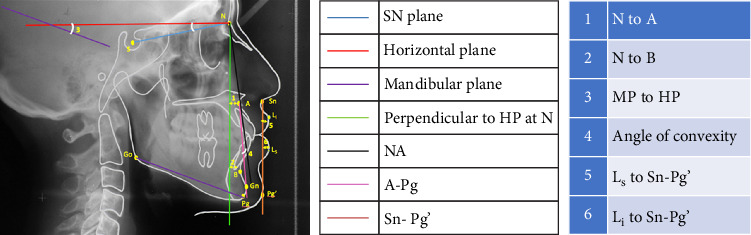
COGS tracing.

**Figure 2:**
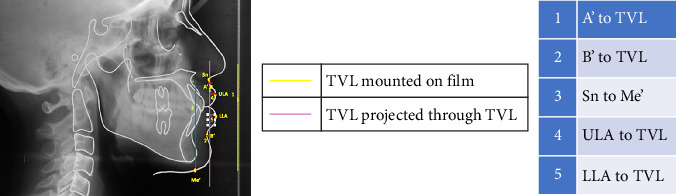
STCA tracing.

All lateral cephalograms were taken on the PLANMECA Proline EC machine with a maximum exposure time of 0.5 s using high-speed polyester-based 18 × 24 cm Kodak X-Omat lateral head films. Metallic markers were placed on the right side of the face to mark key midface structures, as described by Arnett et al. [6] in STCA. The lateral head films were traced for both COGS and STCA analyses (Figures 1 and 2) on an acetate tracing sheet using variable illumination on a view box for the parameters shown in Table 1. Inferences were derived for each variable for each patient according to the norms presented in Table 2. The malocclusion was considered mild when intermaxillary jaw base relationship values were within the normal range, moderate when beyond 1 standard deviation but within 2 standard deviations, and severe when values were beyond 2 standard deviations. Surgical intervention was assumed necessary whenever the malocclusion was inferred severe cephalometrically.

### 2.3. Extraoral Photographs

**Figure 3:**
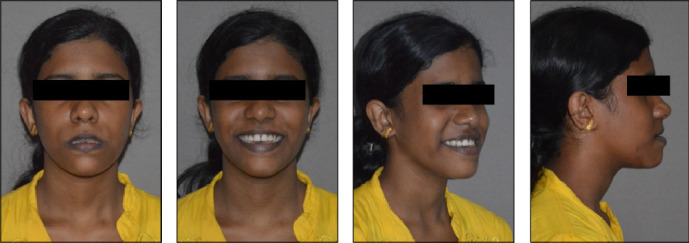
Extraoral photographs.

Four extraoral photographs of these patients were obtained, namely, frontal at rest and at smiling, profile, and three-quarter smiling (Figure 3). The frontal at rest and profile pictures were taken with the natural head position, seated condyles, and passive lips. Smiling photographs were taken with a spontaneous smile so that skeletal malocclusion could be appreciated. All photographs adhered to the American Board of Orthodontics (ABO) standards, with the patient's head oriented accurately in all three planes of space and on the Frankfort horizontal. Ears were exposed for orientation, and eyes were kept open and looking straight ahead; glasses, if any, were removed. Quality lighting revealing facial contours with a white or light background, free of any shadows and distractions, was used. Photographs were maintained at approximately one-quarter life size.

### 2.4. Questionnaire

**Figure 4:**
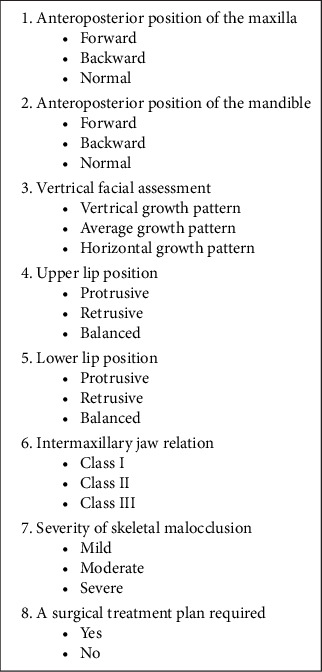
Questionnaire.

The obtained photographs were presented to two experts for their clinical opinion and diagnosis based on parameters mentioned in the questionnaire (Figure 4). The first expert is an orthodontist with a long-standing experience of 15 years in treating patients, while the second is an oral surgeon with extensive experience in orthognathic surgeries. A total of 175 questionnaires were filled out by each expert, ensuring that 120 patients could be included in the study for each variable. Patients were included in the study only when both experts agreed on the diagnosis.

## 3. Statistical Analysis

Kappa analysis was used to calculate the agreement between clinical opinion and the COGS and STCA analyses. Agreement is the degree to which two raters or methods provide consistent results when evaluating the same subjects. Percentage agreement is calculated as the proportion of cases where both methods agree, divided by the total number of cases. Kappa, on the other hand, is a statistical measure that assesses the level of agreement between two categorical variables, adjusting for chance. A kappa value of 0 indicates no agreement beyond chance, while a value of 1 indicates perfect agreement.

Altman's criteria (2011) were referenced to interpret the kappa values. According to Altman, kappa values above 0.40 were considered to provide at least fair agreement, while values above 0.75 were considered to indicate excellent agreement [9].

The level of significance was adjusted according to Bonferroni's correction, and statistical significance was considered when the *p*-value was less than 0.016. All analyses were performed using SPSS software (version 20).

## 4. Results

The study sample consisted of 120 participants, categorized according to their malocclusion types and sagittal skeletal patterns. The characteristics of the sample are summarized in Table 3. Crosstabs of the kappa analysis were evaluated to understand the agreement between clinical opinion with COGS and STCA. The agreement of clinical opinion for the position of the maxilla with COGS and STCA was poor (*K* = negative), with only 55% and 56% agreement, respectively (*p*-value = 0.78). The position of the mandible showed significant values with both STCA and COGS. While both analyses showed fair agreement with clinical opinion, COGS demonstrated a better correlation (*K* = 0.3) than STCA (*K* = 0.2) (Table 4). The agreement in COGS was 58.3%, while that with STCA was 49%. The *p*-value for both analyses was <0.001. The agreement of COGS with clinical opinion when the growth pattern was considered was found to be fair (*K* = 0.2) and significant (*p* < 0.001). A 60% agreement was found. However, the same for STCA was nonsignificant (*p*=0.11) with poor agreement (*K* = 0.1) of 40%. The agreement of upper lip prominence in COGS with clinical opinion was nonsignificant (0.04) with poor agreement (*K* = 0.1). In STCA, the *K* values could not be calculated as the disagreement was large. For lower lip prominence, the agreement of both analyses was poor with COGS and STCA (*K* = 0.01 and 0.02, respectively). However, the correlation of COGS was significant (*p*=0.001). The agreement between clinical diagnosis and both analyses for intermaxillary relationship was significant. COGS showed a moderate agreement of 66% (*K* = 0.4). STCA showed a fair agreement of 62.5% (*K* = 0.35). The *p*-value for both analyses was <0.001. The severity of skeletal malocclusion showed significant results in COGS and nonsignificant for STCA. The agreement was poor for both analyses (with COGS *K* = 0.18, *p*=0.004 and STCA *K* = 0.12, *p*=0.05). The correlation of STCA with a clinical opinion on the need for surgical intervention was fair (*K* = 0.23) and significant (*p*=0.01) with 60.8% agreement. This was better than the correlation seen in COGS (*K* = 0.19), which was poor although significant (*p*=0.01). The concise results have been presented in Table 3. The agreement between COGS and STCA for various measurements was additionally recorded. The agreement between the two analyses was found to be poor for all parameters except for the intermaxillary jaw relationship where the agreement was fair (*K* = 0.2) with a significant value (*p*=0.001) (Table 5).

## 5. Discussion

A total of 175 questionnaires were completed by the orthodontist and oral surgeon. Bell et al. [10] have shown that although oral surgeons and orthodontists evaluate facial profiles similarly, surgeons are more likely to recommend surgical correction. Hence, to remove any specialty bias, only 120 patients out of the 175 questionnaires filled were taken into the study for each parameter,that is, only if the oral surgeon and orthodontist had diagnosed the patient similarly for that parameter.

In a study by Patel and Trivedi [11], they emphasized the use of photographs in a country like India where expensive radiological records might be difficult to obtain. They concluded that photographs may be used reliably for epidemiological purposes, screening, initial consultations, and cases where irradiation is contraindicated or needs to be avoided.

All cephalograms were taken in the more reliable, reproducible natural head position, which uses an extracranial reference line. It is more accurate in representing the realistic appearance of the patient than other reference planes. All cephalogram tracings were done by a single examiner to remove any interexaminer errors [12, 13]. The parameters chosen for tracing were such that each parameter had a corresponding and comparable measurement in both the analyses that were used. For vertical facial assessment in STCA, Sn to Me' was chosen. Nanda [14] established that the anterior dimensions of the face demonstrated typologically divergent patterns of development in open- and deep-bite faces. Hence, facial height, although not the best choice, was taken here to be indicative of the growth pattern.

For Class II and Class III malocclusions requiring orthognathic surgery, several key cephalometric features have been identified. These include increased ANB angles, maxillary retrusion, mandibular retrognathia (Class II) or prognathia (Class III), and significant vertical discrepancies [9]. Both COGS and STCA rely on sagittal evaluations, yet clinicians often consider vertical discrepancies during surgical planning. This was evident in the present study, where STCA demonstrated better predictive accuracy for surgical intervention despite its limitations in growth pattern evaluation.

Analyzing the results for the position of the maxilla, it was seen that the agreement was poor with the clinical opinion for both analyses. In STCA, the maxillary position is measured as the distance from Sn to A'. TVL is placed through subnasale in all cases except maxillary retrusion cases where it is moved 1–3 mm anterior when midface retrusion is diagnosed [15]. In the present study, the TVL was moved 3 mm ahead in all patients where maxillary retrusion was diagnosed clinically. However, the exact amount by which TVL should be moved in which patient is ambiguous as defined in STCA and remains a limitation of this analysis. The TVL line defined in STCA through subnasale may not be a good guide for defining the position of the maxilla as its position corresponds to the position of the maxilla (i.e., it will be forwardly or backwardly placed along with the maxilla) and has been used in many studies as a landmark to measure the growth of the maxilla [16]. The normal values too have only a range of 1.6 mm which may not be clinically very significant.

COGS uses HP as a reference line which is 7° to the SN plane line. Hence, the values vary with the tipping of the anterior cranial base [17] and with the varying linear distance between N and A point. The hard tissue A point is one of the most nonreproducible landmarks in cephalometry. These values hence can be deceptive and might be the reason for the poor agreement between clinical judgment and cephalometric inferences.

The agreement between clinical opinion and cephalometric inferences for the position of the mandible was fair for both analyses. The lower agreement in STCA could be attributed to varying soft tissue thickness in patients which might mask the true discrepancy in the position of the mandible.

The agreement of COGS with clinical opinion for growth pattern was found to be significant with a fair correlation of 60% (Table 4). However, the same for STCA was nonsignificant with a poor correlation (*K* = 0.1) of 40%. Celikoglu et al. [18] used CBCT to assess the soft tissue thickness at the lower anterior face in adult patients with different skeletal vertical patterns. Soft tissue thickness values were found to be the lowest in the high-angle group for both women and men. The soft thickness at the labrale superius, labrale inferius, and pogonion for women was found to be statistically and significantly smaller in the high-angle group compared to values in the normal-angle group. However, for men, no statistically significant differences were found among the vertical growth patterns. This variation can be the reason for the low agreement in STCA. In this study, the sample was not analyzed for gender variations. Future studies can be done to test the agreement in clinical opinion with cephalometric data in males and females separately. Arnett also mentioned that the thickness of the upper lip, lower lip, B to B', Pog to Pog', and Me to Me' alters facial profile. Lower facial esthetic balance is largely controlled by soft tissue thickness in combination with dentoskeletal factors. Hence, soft tissue thickness at Me to Me' may alter the facial height affecting the inference derived for growth pattern in this study. It is to be noted here that the growth pattern inferences are derived from a linear measurement in STCA. No angular measurements (like HP to MP in COGS) or proportions (like Jarabak's ratio) are defined in STCA for diagnosing the growth pattern. This poses a limitation of STCA concerning growth patterns [19].

The agreement of clinical opinion with both analyses for lip prominences was poor. For both upper and lower lips, COGS was better. In both analyses, the measurement here was essentially one of soft tissue. For time and again in the literature, the importance of variability in lip thickness on the profile has been emphasized. Oliver [20] in his study showed a significant correlation between osseous changes and soft tissue changes in both males and females. These correlations were found to be strong in subjects with thin lips. No significant correlations were found in subjects with thick lips. Hence, the lip thickness might mask the skeletal malocclusion and give a variable appearance of prominence. Hence, it can be justified that the agreement in this study was poor for both analyses.

COGS showed a better agreement with clinical opinion than STCA for intermaxillary relationships. The evaluation of the position of the maxilla and mandible in STCA has already been shown to be poorer than that in COGS [21]. The parameter used here for the skeletal discrepancy is A' to B'. Thus, it is predictable that this too shall be poorer than COGS. The angle of convexity proved a good measure of sagittal jaw base discrepancy. The advantages of using the angle of convexity instead of A to B were that errors in occlusal planes could be avoided. Also, no other referencing plane had to be used [22].

The severity of skeletal malocclusion was inferred as severe if the value was traced beyond 2 standard deviations from the norm. As the envelope of discrepancy states, any movement of more than 12 mm can be done only surgically. Hence, the malocclusion when considered severe was inferred to be a surgical case [8]. This study showed STCA to be a better analysis in predicting the surgical requirements of a case. Previous studies [23–25] have suggested that treating patients to dentoskeletal standards may or may not produce desirable esthetics, and the results are seldom predictable. The results in this study too were in accordance.

## 6. Conclusion

The following conclusions can be drawn from the above study:

1. COGS demonstrated better analysis and showed greater agreement with the clinical diagnoses provided by experts.
2. COGS exhibited superior diagnostic accuracy compared to STCA, except in predicting the need for a surgical treatment plan, where STCA appeared to be more effective.
3. No single analysis can be solely relied upon for comprehensive treatment planning. A combination of different cephalometric analyses, clinical findings, and a complete set of diagnostic records should be thoroughly evaluated before confirming a treatment plan.
4. There was poor agreement between COGS and STCA for all parameters, except for the intermaxillary relationship, which showed a fair agreement.

## 7. Limitations

The need for surgery and the severity of malocclusion were assessed cephalometrically only on a sagittal axis, whereas the clinicians considered the vertical discrepancy too while giving their clinical diagnosis. This could have affected the degree of agreement in the study.

## Figures and Tables

**Table 1:** Variables traced.

Clinical parameters	Variables measured in COGS	Variables measured in STCA
Position of the maxilla	N to A	A' to TVL
Position of the mandible	N to B	Sn to Me'
Vertical facial assessment	MP to HP	A' to B'
Intermaxillary jaw relation	Angle of skeletal facial convexity	The distance from ULA to TVL
Upper lip prominence	The distance between *L*_s_ and the line drawn from Sn to Pg'	The distance from ULA to TVL
Lower lip prominence	The distance between *L*_i_ and the line drawn from Sn to Pg'	The distance from LLA to TVL

*Note:* A, point A; A', soft tissue point A; B, point B; Me', soft tissue menton; Pg', soft tissue pogonion.

Abbreviations: HP, horizontal plane; L_i_, labrale inferius; LLA, lower lip anterior; L_s_, labrale superius; MP, mandibular plane; N, nasion; Sn, subnasale; TVL, true vertical line; ULA, upper lip anterior.

**Table 2:** Norms used.

Variable	COGS male norms	COGS female norms	STCA male norm	STCA female norm
Position of the maxilla	0 SD 3.7	−2 SD 3.7	−0.3 SD 1	−0.1 SD 1
Position of the mandible	−5.3 SD 6.7	−6.9 SD −4.3	−7.1 SD 1.6	−5.3 SD 1.5
Growth pattern	24.2 SD 5	24.2 SD 5	81.1 SD 4.7	71.1 SD 3.5
Upper lip prominence	3 SD 1	3 SD 1	3.3 SD 1.7	3.7 SD 1.2
Lower lip prominence	2 SD 1	2 SD 1	1 SD 2.2	1.9 SD 1.4
Intermaxillary relationship	3.9 SD 6.4	2.6 SD 5.1	6.8 SD 1.5	5.2 SD 1.6

**Table 3:** Characteristics of the sample.

Characteristic	Category	No. of patients (*n* = 120)
Malocclusion type	Class I malocclusion	45
	Class II malocclusion	50
	Class III malocclusion	25
Sagittal skeletal pattern	Class I skeletal malocclusion	48
	Class II skeletal malocclusion	53
	Class III skeletal malocclusion	19

**Table 4:** Results in concise.

Parameter	Cephalometric analysis	Percentage agreement	*K* value	*p* value
Position of the maxilla	COGS	66/120 = 55%	−0.02	0.78
	STCA	68/120 = 56.7%	0.09	0.08
Position of the mandible	COGS	70/120 = 58.3%	0.3	<0.001
	STCA	59/120 = 49.1%	0.2	<0.001
Growth pattern	COGS	72/120 = 60%	0.2	<0.001
	STCA	48/120 = 40%	0.07	0.11
Upper lip	COGS	70/120 = 58.3%	0.12	0.04
	STCA	55/120 = 45.8%	—	—
Lower lip	COGS	65/120 = 54.2%	0.17	0.001
	STCA	46/120 = 38.2%	0.02	0.62
Intermaxillary jaw relationship	COGS	79/120 = 65.8%	0.4	<0.001
	STCA	75/120 = 62.5%	0.35	<0.001
Severity of malocclusion	COGS	54/120 = 45%	0.18	0.004
	STCA	51/120 = 42.5%	0.12	0.05
Need for surgical intervention	COGS	76/120 = 63.3%	0.19	0.01
	STCA	73/120 = 60.8%	0.23	0.01

**Table 5:** Agreement between COGS and STCA.

Parameter	Percentage agreement between COGS and STCA	*K* value	*p* value
Position of the maxilla	67/120 = 55.8%	0.18	0.004
Position of the mandible	53/120 = 44.2%	0.14	0.007
Growth pattern	54/120 = 45%	0.12	0.012
Lower lip	39/120 = 32.5%	0.07	0.05
Intermaxillary jaw relationship	66/120 = 55%	0.2	0.001
Severity of malocclusion	39/120 = 32.5%	0.05	0.3
Need for surgical intervention	63/120 = 52.5%	0.11	0.12

## Data Availability

Data are available from the first and corresponding author upon request.

## References

[1] Burstone C. J., James R. B., Legan H., Murphy G. A., Norton L. A. (1978). Cephalometrics for Orthognathic Surgery. *Journal of Oral Surgery*.

[2] Legan H. L., Burstone C. J. (1980). Soft Tissue Cephalometric Analysis for Orthognathic Surgery. *Journal of Oral Surgery*.

[3] Tweed C. H. (1944). Indications for Extraction of Teeth in Orthodontic Procedure. *American Journal of Orthodontics and Oral Surgery*.

[4] Downs W. B. (1956). Analysis of the Dentofacial Profile. *Angle Orthodontist*.

[5] Holdaway R. A. (1956). Changes in Relationship of Points A and B During Orthodontic Treatment. *American Journal of Orthodontics*.

[6] Arnett G. W., Jelic J. S., Kim J. (1999). Soft Tissue Cephalometric Analysis: Diagnosis and Treatment Planning of Dentofacial Deformity. *American Journal of Orthodontics and Dentofacial Orthopedics*.

[7] Borzabadi-Farahani A., Olkun H. K., Eslamian L., Eslamipour F. (2024). A Retrospective Investigation of Orthognathic Patients and Functional Needs. *Australasian Orthodontic Journal*.

[8] Mankad B., Cisneros G. J., Freeman K., Eisig S. B. (1999). Prediction Accuracy of Soft Tissue Profile in Orthognathic Surgery. *International Journal of Adult Orthodontics and Orthognathic Surgery*.

[9] Borzabadi-Farahani A., Borzabadi-Farahani A. (2011). Agreement Between the Index of Complexity, Outcome, and Need and the Dental and Aesthetic Components of the Index of Orthodontic Treatment Need. *American Journal of Orthodontics and Dentofacial Orthopedics*.

[10] Bell R., Kiyak H. A., Joondeph D. R., McNeill R. W., Wallen T. R. (1985). Perceptions of Facial Profile and Their Influence on the Decision to Undergo Orthognathic Surgery. *American Journal of Orthodontics*.

[11] Patel D. P., Trivedi R. (2013). Photography Versus Lateral Cephalogram: Role in Facial Diagnosis. *Indian Journal of Dental Research*.

[12] Lundström A., Lundström F., Lebret L. M., Moorrees C. F. (1995). Natural Head Position and Natural Head Orientation: Basic Considerations in Cephalometric Analysis and Research. *The European Journal of Orthodontics*.

[13] Lundström F., Lundström A. (1992). Natural Head Position as a Basis for Cephalometric Analysis. *American Journal of Orthodontics and Dentofacial Orthopedics*.

[14] Nanda S. K. (1988). Patterns of Vertical Growth in the Face. *American Journal of Orthodontics and Dentofacial Orthopedics*.

[15] Sabri R. (2005). The Eight Components of a Balanced Smile. *Journal of Clinical Orthodontics: JCO*.

[16] Woodside D. G., Linder-Aronson S., Lundström A., McWilliam J. (1991). Mandibular and Maxillary Growth After Changed Mode of Breathing. *American Journal of Orthodontics and Dentofacial Orthopedics*.

[17] Jacobson A. (1976). Application of the “Wits” Appraisal. *American Journal of Orthodontics*.

[18] Celikoglu M., Buyuk S. K., Ekizer A., Sekerci A. E., Sisman Y. (2015). Assessment of the Soft Tissue Thickness at the Lower Anterior Face in Adult Patients With Different Skeletal Vertical Patterns Using Cone-Beam Computed Tomography. *The Angle Orthodontist*.

[19] Kolokitha O.-E., Chatzistavrou E. (2012). Factors Influencing the Accuracy of Cephalometric Prediction of Soft Tissue Profile Changes Following Orthognathic Surgery. *Journal of Maxillofacial and Oral Surgery*.

[20] Oliver B. M. (1982). The Influence of Lip Thickness and Strain on Upper Lip Response to Incisor Retraction. *American Journal of Orthodontics*.

[21] Park Y.-C., Burstone C. J. (1986). Soft-Tissue Profile-Fallacies of Hard-Tissue Standards in Treatment Planning. *American Journal of Orthodontics and Dentofacial Orthopedics*.

[22] Friede H., Kahnberg K.-E., Adell R., Ridell A. (1987). Accuracy of Cephalometric Prediction in Orthognathic Surgery. *Journal of Oral and Maxillofacial Surgery*.

[23] Kocadereli I. (2002). Changes in Soft Tissue Profile After Orthodontic Treatment With and Without Extractions. *American Journal of Orthodontics and Dentofacial Orthopedics*.

[24] Stellzig-Eisenhauer A., Lux C. J., Schuster G. (2002). Treatment Decision in Adult Patients With Class III Malocclusion: Orthodontic Therapy or Orthognathic Surgery?. *American Journal of Orthodontics and Dentofacial Orthopedics*.

[25] Bryan D. C., Hunt N. P. (1993). Surgical Accuracy in Orthognathic Surgery. *British Journal of Oral and Maxillofacial Surgery*.

